# The complete chloroplast genome of *Calanthe arcuata,* an endemic terrestrial orchid in China

**DOI:** 10.1080/23802359.2019.1639561

**Published:** 2019-07-18

**Authors:** Hui Zhong, Li-Ming Shen, Hai-Ping Liu, Zhong-Jian Liu, Sha-Sha Wu, Jun-Wen Zhai

**Affiliations:** a Key Laboratory of National Forestry and Grassland Administration for Orchid Conservation and Utilization at College of Landscape Architecture, Fujian Agriculture and Forestry University, Fuzhou, China;; b Fujian Ornamental Plant Germplasm Resources Innovation and Engineering Application Research Center, Fuzhou, China

**Keywords:** *Calanthe arcuata*, chloroplast genome, Orchidaceae, Illumina sequencing, phylogenetic

## Abstract

*Calanthe arcuata* is an endemic terrestrial orchid in China with high value of ornament and breeding. Here, we reported the first complete chloroplast genome of this plant in this research. The circular genome is 158,735 bp in length and includes large single-copy (LSC) region of 87,348 bp, small single-copy (SSC) region of 18,489 bp, and a pair of invert repeats (IR) regions of 26,449 bp. It contains 136 genes, including 88 protein-coding (PCG), 38 transfer RNA (tRNA), and eight ribosomal RNA (rRNA) gene. The phylogenetic analysis indicated that *C. arcuata* is the sister to *C. davidii* and *C. triplicata*.

*Calanthe arcuata* is an endemic terrestrial orchid widely distributed in China (Gansu, Hubei, Shaanxi, Guizhou, Sichuan, Yunnan, Tibet, and Taiwan) (Chen et al. 2010). *Calanthe arcuata* usually grows in soil or rocks along streams in humus-rich valleys, under mixed forests at altitudes between 2700 and 3450 m (Clayton and Cribb 2013). The distribution of this species has the characteristics of continental and island discontinuous distribution. Due to climate change, human destruction, and habitat loss or fragmentation, wild populations of *C. arcuata* sharply decreased in recent years. As a result, this species has been listed as a vulnerable species in the Red List (IUCN 2018). In order to better protection of this orchid and understand their genetic information, we assembled complete chloroplast genome data, which would be helpful for the population genetic and phylogenetic research of *C. arcuata*.

We assembled the complete chloroplast genome of *C. arcuata* using a silicon dried sample, which was collected from Taibai Mountain, Shaanxi, China (N 34°05′, E 107°75′). DNA was stored at Fujian Agriculture and Forestry University (specimen voucher S69). The total genomic DNA was extracted using a modified CTAB method (Doyle and Doyle 1987) and sequenced based on the Illumina pair-end technology. The clean reads were firstly aligned to *C. triplicata* (GenBank accession No. KF753635) (Yang et al. 2014). Filtered reads were then assembled into contigs in CLC Genomics Workbench version 8.0 (CLC Bio, Aarhus, Denmark). The genome was automatically annotated using DOGMA (Wyman et al. 2004), then adjusted using Geneious version 11.1.15 (Kearse et al. 2012) and submitted to GenBank with accession number MK934523. The complete chloroplast genome of *C. arcuata* is 158,735 base pairs (bp) in length, containing a large single-copy (LSC) region of 87,348 bp, a small single-copy (SSC) region of 18,489 bp, and two inverted repeat (IR) regions of 26,449 bp. A total of 136 genes were annotated, including 88 protein-coding (PCG), 38 transfer RNA (tRNA), and eight ribosomal RNA (rRNA) gene. The complete genome GC content was 36.60%, whereas the corresponding values of the LSC, SSC, and IR regions are 34.2%, 29.7%, and 43%, respectively.

In order to study the phylogenetic position of *C. arcuata*, a phylogenetic analysis was carried out with 22 complete chloroplast genomes of Orchidaceae species, which included 18 Epidendroideae species (*Bletilla ochracea, B. striata, Calanthe davidii, C. triplicata, Cattleya crispata, C. liliputana, Cremastra appendiculata, Cymbidium aloifolium, C. ensifolium, C. mannii, C. tracyanum, Dendrobium catenatum, D. nobile, Epipactis mairei, Erycina pusilla, Gastrochilus fuscopunctatus, Pleione bulbocodioides,* and *Sobralia callosa*) and four Orchidoideae species (*Goodyera fumata*, *Anoectochilus emeiensis*, *Ludisia discolor* and *Habenaria radiata*) as outgroup. All of the data was downloaded from NCBI GenBank. The sequences were aligned using HomBlocks pipeline (Bi et al. 2017). RAxML-HPC2 on XSEDE version 8.2.10 (Stamatakis 2014) was used to construct a maximum likelihood tree, the branch support was computed with 1,000 bootstrap replicates. The ML tree analysis indicated that *C. arcuata* is sister to *C. davidii* (MG925365) and *C. triplicata* (KF753635) with 100% bootstrap support (Figure 1).

**Figure 1.**
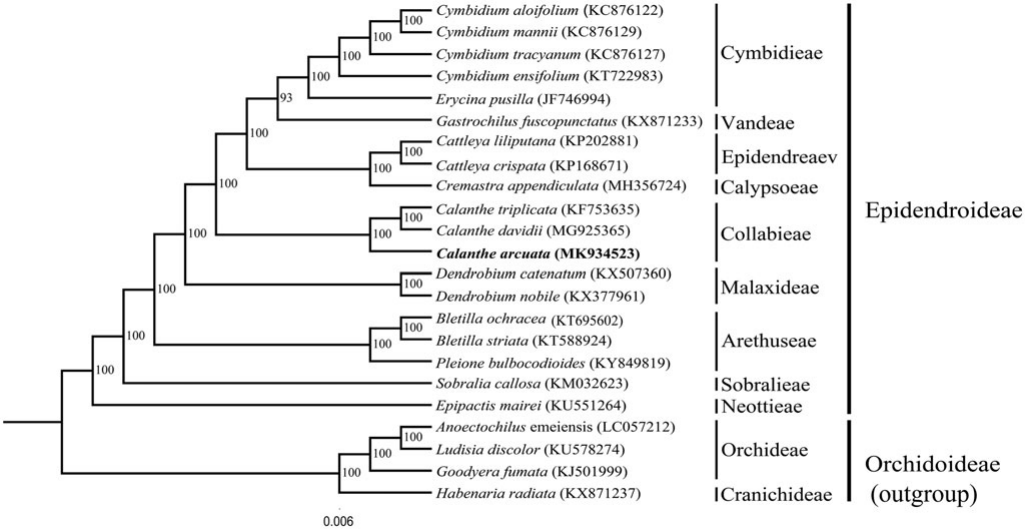
The maximum-likelihood (ML) tree based on 18 species of Epidendroideae, based on whole chloroplast genome sequences, with Orchidoideae: *Goodyera fumata*, *Anoectochilus emeiensis*, *Ludisia discolor*, and *Habenaria radiata* as outgroup. The bootstrap value based on 1000 replicates is shown on each node, and the position of *Calanthe arcuata* is shown in bold.
